# Retroperitoneal cutaneous ureterostomy following radical cystectomy: A multicenter comparative study of robotic versus open surgery

**DOI:** 10.1111/iju.15580

**Published:** 2024-09-10

**Authors:** Yutaro Sasaki, Kyotaro Fukuta, Fumiya Kadoriku, Kei Daizumoto, Keito Shiozaki, Ryotaro Tomida, Yoshito Kusuhara, Tomoya Fukawa, Yutaka Yanagihara, Ryoichi Nakanishi, Kunihisa Yamaguchi, Yasuyo Yamamoto, Hirofumi Izaki, Masayuki Takahashi, Kenjiro Okamoto, Junya Furukawa

**Affiliations:** ^1^ Department of Urology Tokushima University Graduate School of Biomedical Sciences Tokushima Japan; ^2^ Department of Urology Tokushima Prefectural Central Hospital Tokushima Japan; ^3^ Department of Urology Ehime Prefectural Central Hospital Matsuyama Japan

**Keywords:** open radical cystectomy, retroperitoneal cutaneous ureterostomy, robot‐assisted radical cystectomy

## Abstract

**Introduction:**

The aim of this study was to evaluate the differences in perioperative outcomes of cutaneous ureterostomy (CUS) between open surgery (open radical cystectomy, ORC) and robot‐assisted surgery (robot‐assisted radical cystectomy, RARC), including the stent‐free rate, readmission rates due to urinary tract infection (UTI), and changes in renal function.

**Methods:**

Between 2005 and 2023, a total of 37 patients underwent CUS following ORC, while 24 patients underwent CUS following RARC. Perioperative outcomes were compared between these two groups.

**Results:**

The patients in the RARC group were significantly older (*p* = 0.007) and had a significantly higher proportion of high‐risk cases with ASA‐PS ≥3 (*p* = 0.002). In addition, RARC was associated with a significantly lower estimated blood loss (*p* < 0.001) and a reduced transfusion rate (*p* = 0.003). Postoperative complication rates and the stent‐free rate were comparable between the ORC and RARC groups. Throughout a median follow‐up period of 2.6 years, rates of readmission due to UTI did not differ significantly between the two groups. Moreover, there were no differences in the change in estimated glomerular filtration rate before and after surgery and the 3‐year survival rates were similar across both groups.

**Conclusions:**

CUS following RARC appears to offer a safer alternative compared with CUS following ORC, and the stent‐free rates are comparable. The significantly lower estimated blood loss and transfusion rate associated with RARC are particularly favorable for elderly patients, those who are frail, and individuals with multiple comorbidities.

**Abbreviations & Acronyms**
ASA‐PS: American Society of Anesthesiologists physical status classification
CSS: cancer‐specific survival
CUS: cutaneous ureterostomy
eGFR: estimated glomerular filtration rate
IQR: interquartile range
ORC: open radical cystectomy
OS: overall survival
POM: postoperative month
RARC: robot‐assisted radical cystectomy
RFS: recurrence‐free survival
UTI: urinary tract infection

## BACKGROUND

Compared with open radical cystectomy (ORC), robot‐assisted radical cystectomy (RARC) offers enhanced surgical and oncological safety and efficacy, making it a more acceptable option for elderly patients, frail individuals, and those with significant comorbidities.^1^ With an increasing number of these patients requiring radical cystectomy, there has been renewed interest in cutaneous ureterostomy (CUS), which is associated with a lower risk of perioperative complications.^2^ However, there is a lack of literature comparing open and robot‐assisted approaches in retroperitoneal CUS following radical cystectomy.^3^ Therefore, this study aims to evaluate the perioperative outcomes of CUS between open surgery and robot‐assisted surgery; focusing on parameters such as stent‐free rate, rates of readmission due to urinary tract infection (UTI), and changes in renal function. This study also aims to evaluate the oncological outcomes of CUS, including overall survival (OS), cancer‐specific survival (CSS), and recurrence‐free survival (RFS) between the two groups.

## METHODS

**FIGURE 1:**
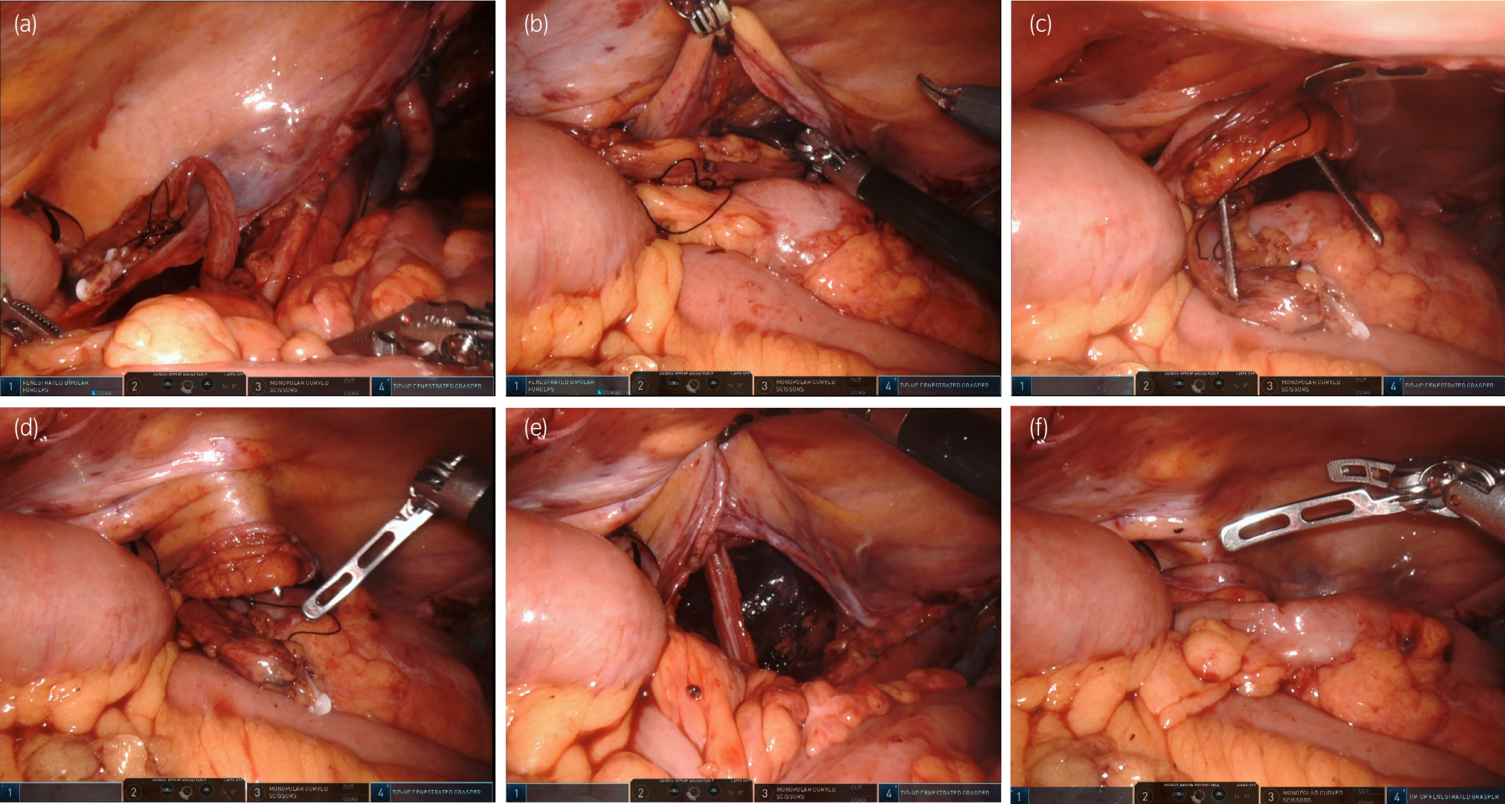
Surgical images showing retroperitoneal CUS following robot‐assisted radical cystectomy (RARC). (a) The console surgeon dissects the left ureter toward the kidney to prevent any potential bending or stretching. (b) The console surgeon then expands the retroperitoneal space laterally toward the stoma site. (c) The patient‐side surgeon expands the retroperitoneal space using Pean forceps. (d) The patient‐side surgeon retracts the left ureter out of the body. (e) The console surgeon checks to make sure the left ureter is not bent. (f) Retroperitonealization of the left ureter is completed. The stoma is then created using the Toyoda method.

For this retrospective cohort study, we conducted a thorough review of medical records from three hospitals: Tokushima University Hospital, Tokushima Prefectural Central Hospital, and Ehime Prefectural Central Hospital. The study period spanned from June 2005 to July 2023, encompassing a total of 374 patients who underwent radical cystectomy. Among these 374 patients, 109 underwent ORC, and 265 patients underwent RARC. Furthermore, 37 out of the 109 ORC patients and 24 out of the 265 RARC patients underwent retroperitoneal CUS (Table S1). The decision to perform CUS was guided by several factors, including the presence of comorbidities, history of abdominal radiotherapy, and patient preferences. This study compared several perioperative variables, including patient characteristics, and surgical and oncological outcomes between the ORC and RARC groups. Neoadjuvant chemotherapy consisted of 2–4 cycles of either methotrexate, vinblastine, doxorubicin, and cisplatin, or gemcitabine and cisplatin, or gemcitabine and carboplatin. Complications were categorized in accordance with the Clavien–Dindo classification system. Grade ≥ 3 complications were defined as major complications, and grade ≤ 2 complications were defined as minor complications. The surgical procedure for CUS involves several critical steps. Following radical cystectomy, the surgeon first dissects the ureter toward the kidney to prevent any potential bending or stretching. Subsequently, the retroperitoneal space is expanded laterally toward the stoma site. In cases of ipsilateral CUS, where the left ureter is involved, it is tunneled behind the sigmoid colon. Using Pean forceps inserted retroperitoneally from the stoma site, the surgeon carefully retracts the ureter out of the body. In the RARC group, the console surgeon and the patient‐side surgeon collaborate to perform the same steps as in the ORC group (Figure 1). Due to the characteristics of robot‐assisted surgery, it is difficult for the console surgeon to expand the retroperitoneal space near the stoma site, so the patient‐side surgeon expands the retroperitoneal space using Pean forceps or a trocar. The stoma is created using the Toyoda method in the both groups.^4^ Postoperatively, a single J stent (Bander Ureteral Diversion Stent, 6 Fr, 75 cm; Cook Medical, Bloomington, IN, USA) remains in place for 2–4 weeks after surgery. The decision to remove the stent after surgery and perform a stent‐free trial was at the discretion of the operating surgeon. If recurrent UTIs or decreased renal function occurred after the stent‐free trial, the patient was considered for stent reinsertion. Patients who did not require stent reinsertion after the stent‐free trial were defined as “stent‐free.”

### Statistical analyses

All continuous variables are expressed as a median [interquartile range]. Continuous variables were evaluated using student's t‐test for data with normal distribution or the Mann–Whitney U test for data with non‐normal distribution. Nominal variables were compared using Pearson's chi‐square test. Kaplan–Meier analysis with log‐rank test was utilized to compare the OS, CSS, and RFS rates between the two groups. A *p* value of <0.05 was considered statistically significant. All statistical analyses were conducted using EZR (Saitama Medical Center, Jichi Medical University, Saitama, Japan), a graphical user interface for R (ver.4.1.2, www.r‐project.org).

## RESULTS

Table 1 compares the characteristics of the patients between the ORC and RARC groups. Patients in the RARC group were significantly older (median [interquartile range]: 69 [64–77] vs. 75 [71–82] years, *p* = 0.007) and had a higher proportion of high‐risk cases (American Society of Anesthesiologists physical status classification (ASA‐PS) ≥3) compared with the ORC group (14% vs. 54%, *p* = 0.002). There were no significant differences between the two groups in clinical tumor stage or clinical nodal stage. There was no significant difference observed in the rate of lymph node dissection (LND) (76% vs. 88%, *p* = 0.421), but there was a significant difference in the extent of LND performed (*p* < 0.001). In the ORC group, 24 out of the 28 patients underwent limited LND (confined to the obturator and/or perivesical fossa), whereas in the RARC group, 17 out of the 21 patients underwent standard LND (performed up to the common iliac arteries), or extended LND (performed up to the proximal boundary of the crossing of the common iliac vessels with the ureters or the aortic bifurcation).

**TABLE 1:** Comparison of patient characteristics between the ORC and robot‐assisted radical cystectomy (RARC) groups.

	ORC	RARC	*p* value
	(*n* = 37)	(*n* = 24)	
Age, years, median (IQR)	69 (64–77)	75 (71–82)	0.007
Male, *n* (%)	27 (73)	19 (79)	0.807
BMI, kg/m^2^, median (IQR)	23.0 (21.3–25.4)	23.5 (22.3–25.5)	0.555
ASA‐PS ≥3, *n* (%)	5 (14)	13 (54)	0.002
Clinical tumor stage, *n* (%)			
cTis/T1	13 (35)	5 (21)	0.358
cT2	15 (41)	14 (58)	
cT3/T4	9 (24)	5 (21)	
Clinical nodal stage ≥1, *n* (%)	5 (14)	1 (4)	0.449
History of abdominal surgery, *n* (%)	20 (54)	9 (38)	0.316
History of pelvic radiotherapy, *n* (%)	1 (3)	2 (8)	0.698
Solitary kidney, *n* (%)	13 (35)	4 (17)	0.201
Bilateral CUS, *n* (%)	15 (41)	8 (33)	0.766
LND, *n* (%)	28 (76)	21 (88)	0.421
Limited LND, *n* (%)	24 (65)	4 (17)	<0.001
Standard or extended LND, *n* (%)	4 (11)	17 (71)	
Neoadjuvant chemotherapy, *n* (%)	9 (24)	9 (38)	0.415
Adjuvant chemotherapy, *n* (%)	10 (27)	3 (13)	0.301

Abbreviations: ASA‐PS, American Society of Anesthesiologists physical status classification; BMI, body mass index; IQR, interquartile range; LND, lymph node dissection.

**FIGURE 2:**
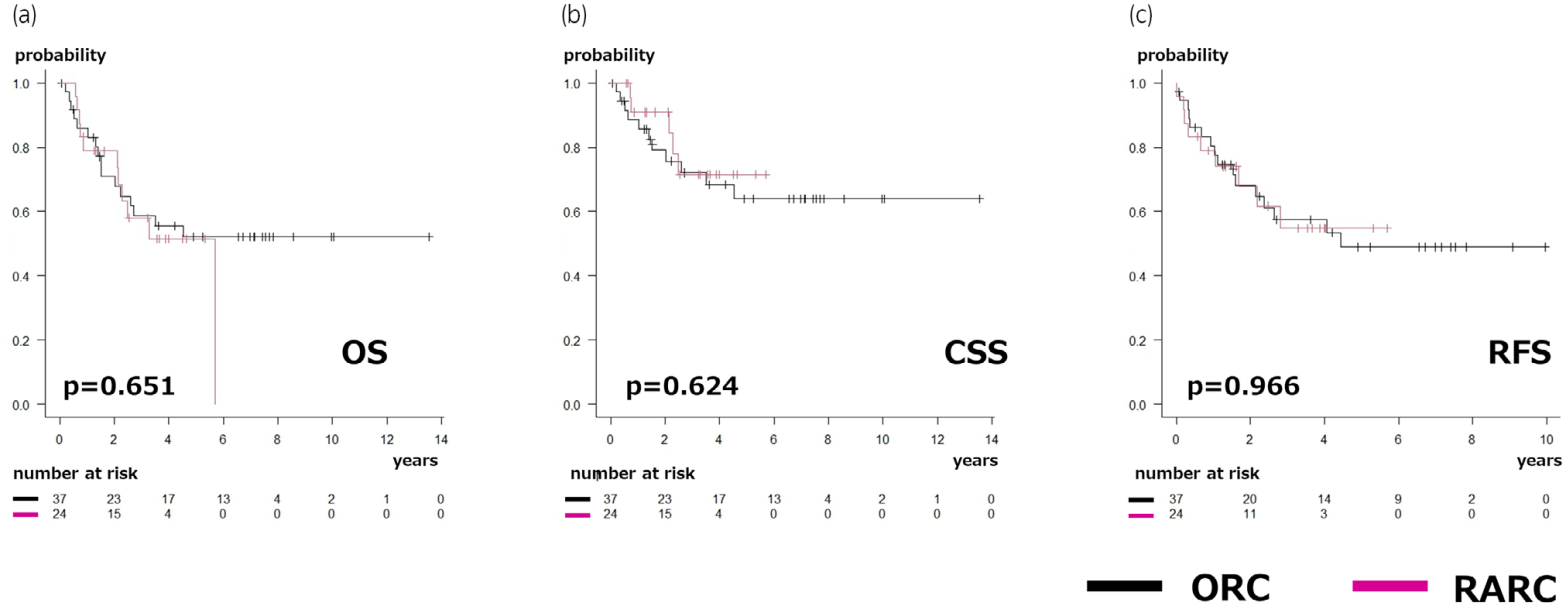
Kaplan–Meier curves of the ORC and robot‐assisted radical cystectomy (RARC) groups. The black line represents patients who underwent ORC, and the pink line represents those who underwent RARC. (a) OS (log‐rank, *p* = 0.651). (b) CSS (log‐rank, *p* = 0.624). (c) RFS (log‐rank, *p* = 0.966). ORC, open radical cystectomy; RARC, robot‐assisted radical cystectomy; OS, overall survival; CSS, cancer‐specific survival; RFS, recurrence‐free survival.

Table 2 presents a comparison of surgical outcomes between the ORC and RARC groups. Operative time did not differ significantly between the groups (382 [330–474] vs. 376 [336–467] mL, respectively; *p* = 0.721). However, the RARC group exhibited significantly lower estimated blood loss (1350 [970–2590] vs. 300 [160–480] mL, respectively; *p* < 0.001) and significantly reduced transfusion rate (76% vs. 33%, *p* = 0.003) compared with the ORC group. Additionally, the RARC group showed a significantly higher lymph node yield (8 [4–12] vs. 13 [9–24], respectively; *p* < 0.001). The incidence of postoperative complications was similar between the ORC and RARC groups. Major complications within 30 days after surgery in the ORC group included ileus (grade 3a), wound dehiscence (grade 3b), and rectal injury (grade 3b) in one patient each. In the RARC group, port site hernia (grade 3b), ileus (grade 3a), and rectal injury (grade 3b) in one patient each. Major complications within 30–90 days after surgery in the ORC group included ileus (grade 3a) in one patient each. In the RARC group, acute myocardial infarction (grade 3a) and UTI (grade 4a) in one patient each. Stent‐free rates showed no significant difference between the two groups (38% vs. 33%, respectively; *p* = 0.932). Over a median follow‐up period of 2.6 years for all patients, the rate of readmission due to UTI was comparable between the ORC and RARC groups (27% vs. 46%, respectively; *p* = 0.279). Table 3 shows the preoperative and postoperative changes in estimated glomerular filtration rate (eGFR). There was no statistically significant difference observed in the change in eGFR between the two groups. Furthermore, the 3‐year survival rates did not differ between the ORC and RARC groups with respect to OS (59% vs. 58%, respectively; *p* = 0.651), CSS (72% vs. 72%, respectively; *p* = 0.624), and RFS (58% vs. 55%, respectively; *p* = 0.966) (Figure 2).

**TABLE 2:** Comparison of surgical outcomes between the ORC and robot‐assisted radical cystectomy (RARC) groups.

	ORC	RARC	*p* value
	(*n* = 37)	(*n* = 24)	
Operative time, min, median (IQR)	382 (330–474)	376 (336–467)	0.721
Estimated blood loss, mL, median (IQR)	1350 (970–2590)	300 (160–480)	<0.001
Transfusion, *n* (%)	28 (76)	8 (33)	0.003
Pathological tumor stage, *n* (%)			
pT0	3 (8)	5 (21)	0.456
pTa/Tis/T1	10 (27)	7 (29)	
pT2	8 (22)	3 (13)	
pT3/T4	16 (43)	9 (38)	
Lymph node yield, median (IQR)	8 (4–12)	13 (9–24)	<0.001
Positive lymph node, *n* (%)	6 (16)	6 (25)	0.608
30‐d complication			
Minor complication, *n* (%)	15 (41)	12 (50)	0.644
Major complication, *n* (%)	3 (8)	3 (13)	0.902
90‐d complication			
Minor complication, *n* (%)	5 (14)	3 (13)	1.000
Major complication, *n* (%)	1 (3)	2 (8)	0.698
Stent‐free rate, *n* (%)	14 (38)	8 (33)	0.932
Follow‐up, years, median (IQR)	3.5 (1.3–7.1)	2.4 (1.2–3.7)	0.204
Readmission due to UTI, *n* (%)	10 (27)	11 (46)	0.279

Abbreviations: IQR, interquartile range; UTI; urinary tract infection.

**TABLE 3:** Changes in eGFR before and after surgery between the ORC and robot‐assisted radical cystectomy (RARC) groups.

	ORC	RARC	*p* value
eGFR, mL/min/1.73 m^2^, median (IQR)			
Preoperative	56.8 (45.0–64.1)	43.5 (34.5–62.5)	0.101
POM 3	49.5 (41.4–62.6)	45.5 (35.0–54.0)	0.317
POM 6	48.0 (34.5–56.6)	39.0 (31.0–58.0)	0.450
POM 12	48.0 (36.0–60.4)	38.0 (31.0–52.0)	0.129
POM 24	44.0 (32.0–54.0)	39.5 (31.0–50.0)	0.590

Abbreviations: eGFR, estimated glomerular filtration rate; IQR, interquartile range; POM, postoperative month.

## DISCUSSION

Urinary diversion following radical cystectomy poses a significant risk of perioperative complications, which is particularly concerning for elderly patients, frail patients, and those with multiple comorbidities.^5^ Among the options available, CUS stands out as the simplest form of urinary diversion that does not involve using intestinal segments. Compared with diversions that utilize intestinal segments, CUS offers advantages such as reduced invasiveness, fewer gastrointestinal complications, shorter operative times, less blood loss, and shorter hospital stays.^2^, ^6^, ^7^ Given the increasing number of elderly patients, frail individuals, and patients with multiple comorbidities requiring radical cystectomy, there has been renewed interest in CUS because of its lower perioperative complication risk.^2^ Compared with ORC, RARC, known for its enhanced surgical and oncological safety and efficacy, is particularly suitable for these patient populations.^1^ Therefore, combining RARC with CUS represents a minimally invasive surgical approach for treating bladder cancer.

Currently, there are no studies comparing the perioperative and oncological outcomes of open surgery and robot‐assisted surgery specifically in patients undergoing CUS. In this study, patients who underwent RARC were significantly older and had a significantly higher proportion of high‐risk cases (ASA PS ≥ 3). However, data regarding screening tools to assess the general condition of elderly patients, such as the G8 and the Flemish version of the Triage Risk Screening Tool, were not available. Operative time did not differ significantly between the two groups, contrary to the anticipated shorter duration with RARC. This similarity in operative times can be attributed to two main factors. First, patients undergoing RARC typically required significantly more extensive LND. Second, performing CUS in robotic‐assisted surgery presents challenges. These include difficulties in dissecting the ureter toward the kidney and expanding the retroperitoneal space, largely due to limitations in the instrument's range of motion and potential interference between robotic arms. The limitations in instrument range of motion and potential interference between robotic arms pose challenges for the console surgeon in expanding the retroperitoneal space during RARC. Similarly, pulling the ureter out of the body from the stoma site can be difficult for the patient‐side surgeon. In contrast, open surgery makes performing CUS relatively easy. Thus, we attribute the prolonged operative time in the RARC group to the extensive LND required and the difficulties associated with CUS in robot‐assisted procedures. Ideally, direct comparisons of the time required for radical cystectomy and CUS between ORC and RARC groups would provide valuable insights. However, the lack of these data in the ORC group precluded such comparisons in this study. Nevertheless, the significantly lower estimated blood loss and transfusion rate in the RARC group are favorable results, particularly beneficial for elderly patients, frail individuals, and patients with multiple comorbidities. In the present study, the overall stent‐free rate following CUS was 36.1%, a result that was similar between the ORC and RARC groups, but falls short of being considered satisfactory. Many studies focusing on CUS have explored surgical techniques aimed at improving the stent‐free rate, reporting favorable results, with rates ranging from 77.8% to 92.4%.^8^, ^9^, ^10^ These results, however, were obtained through open surgery performed by experienced urologists at a high‐volume center, and should be interpreted cautiously. Regarding CUS in robot‐assisted surgery, Tanaka et al. reported three cases of complete retroperitoneal CUS with a median procedure, and all 3 cases achieved stent‐free.^11^ In the present study, our study found that CUS was performed in 33.9% of ORC patients compared with only 9.1% of RARC patients. Because cases of patients who undergo CUS following RARC are less common, accruing sufficient CUS cases poses a challenge. Moving forward, addressing these challenges and refining surgical techniques for CUS in robot‐assisted surgery is crucial to shorten the learning curve and improve stent‐free rates. On the contrary, Nabavizadeh et al. reported the perioperative outcomes of 31 patients who underwent CUS at the Mayo Clinic.^5^ Their strategy involved routinely placing ureteral stents to maintain ureteral patency postoperatively.^5^ Regular exchange of these stents helps prevent ureteral obstruction following surgery. Preserving renal function postoperatively is crucial for elderly patients, frail individuals, and patients with multiple comorbidities. Performing CUS with the intention of maintaining stents offers certain advantages.

The present study has several limitations that warrant consideration. First, it was a retrospective study conducted across three centers with a relatively small sample size, potentially limiting the generalizability of the findings to broader patient populations. Second, differences in patient characteristics, such as age and ASA‐PS, between the ORC and RARC groups may have an influenced the outcomes. Comparisons between the two groups using propensity score matching analysis or multivariate analysis with a larger sample size will be necessary. Third, there were disparities in the inclusion criteria for CUS between the ORC and RARC groups. As mentioned previously, CUS was performed in 33.9% of ORC patients compared with only 9.1% of the RARC group. Given that intracorporeal urinary diversion using intestinal segments is more common following RARC, the smaller number of CUS cases after RARC complicates future case accumulation. Fourth, unclear criteria for stent reinsertion may have influenced the stent‐free rates in the study. Finally, the variability in surgical experience among the participating surgeons might have introduced inconsistencies in both surgical techniques and oncological outcomes.

In conclusion, CUS following RARC appears to offer safer outcomes compared with CUS following ORC, with comparable stent‐free rates. The significantly lower estimated blood loss and transfusion rates associated with RARC are particularly favorable for elderly, frail, and comorbid patients.

## AUTHOR CONTRIBUTIONS

All authors agree with the content of the manuscript. In accordance with the latest guidelines of the International Committee of Medical Journal Editors, each author's contribution to the paper is as follows. **Y.S.:** Project development, data collection, data analysis, and manuscript writing. **K.F.:** Project development, data collection, and data analysis. **F.K.:** Project development, data collection. **K.D.:** Project development, data collection, and data analysis. **K.S.:** Data collection. **R.T.:** Data collection. **Y.K.:** Data collection. **T.F.:** Manuscript writing'editing. **Y. Yanagihara:** Data collection. **R.N.:** Data collection. **K.Y.:** Data collection. **Y. Yamamoto:** Data collection. **H.I.:** Data analysis. **M.T.:** Project development and manuscript writing'editing. **K.O.:** Data collection. **J.F.:** Project development and manuscript writing/editing.

## CONFLICT OF INTEREST STATEMENT

Junya Furukawa is an Editorial Board member of the International Journal of Urology and a co‐author of this article. To minimize bias, they were excluded from all editorial decision‐making related to the acceptance of this article for publication.

## APPROVAL OF THE RESEARCH PROTOCOL BY AN INSTITUTIONAL REVIEWER BOARD

The protocol for this research project has been approved by a suitably constituted Ethics Committee of the institution, and it conforms to the provisions of the Declaration of Helsinki, the Ethics Committee of Tokushima University, Approval No. 4457, the Ethics Committee of Tokushima Prefectural Central Hospital, Approval No. 20–37, and the Ethics Committee of Ehime Prefectural Central Hospital, Approval No. 31–33.

## INFORMED CONSENT

All patients provided written informed consent for treatment and publication of this article.

## REGISTRY AND THE REGISTRATION NO. OF THE STUDY/TRIAL

N/A.

## ANIMAL STUDIES

N/A.

## FUNDING INFORMATION

All authors declare that they have no sources of funding for this research.

## Supporting information


Table S1.
